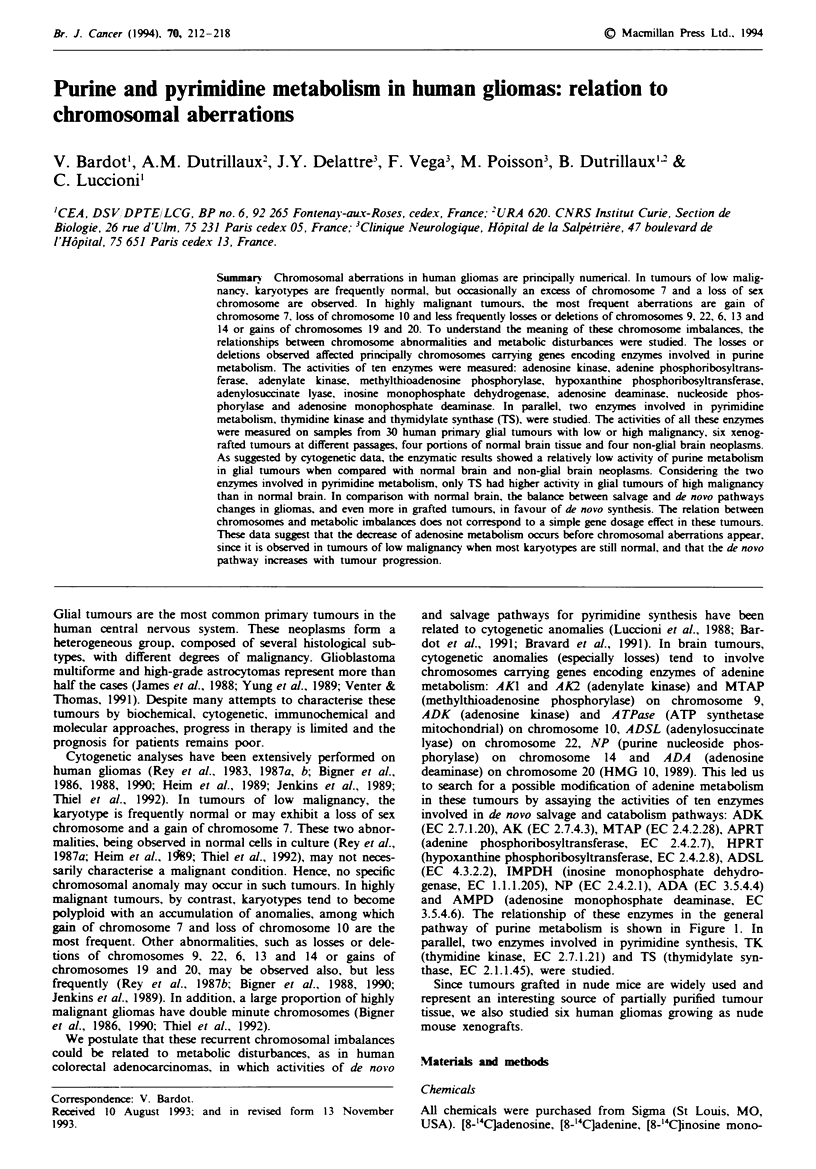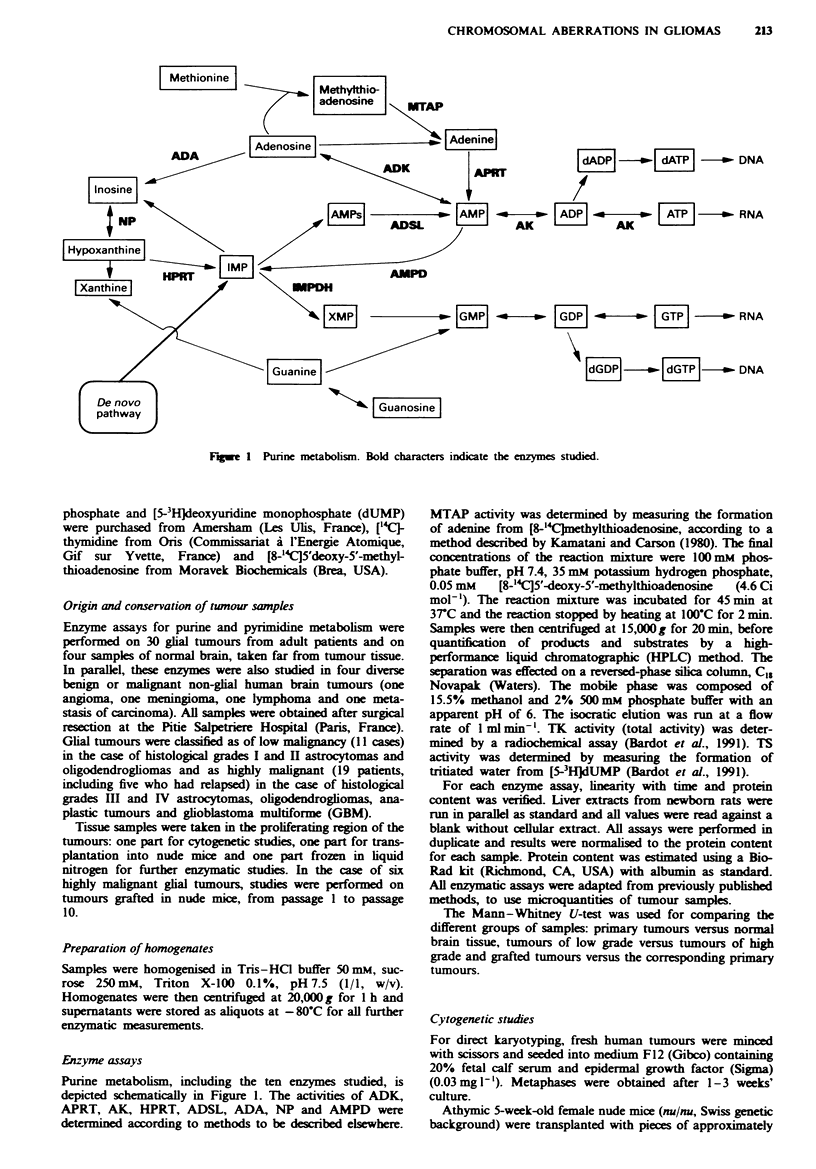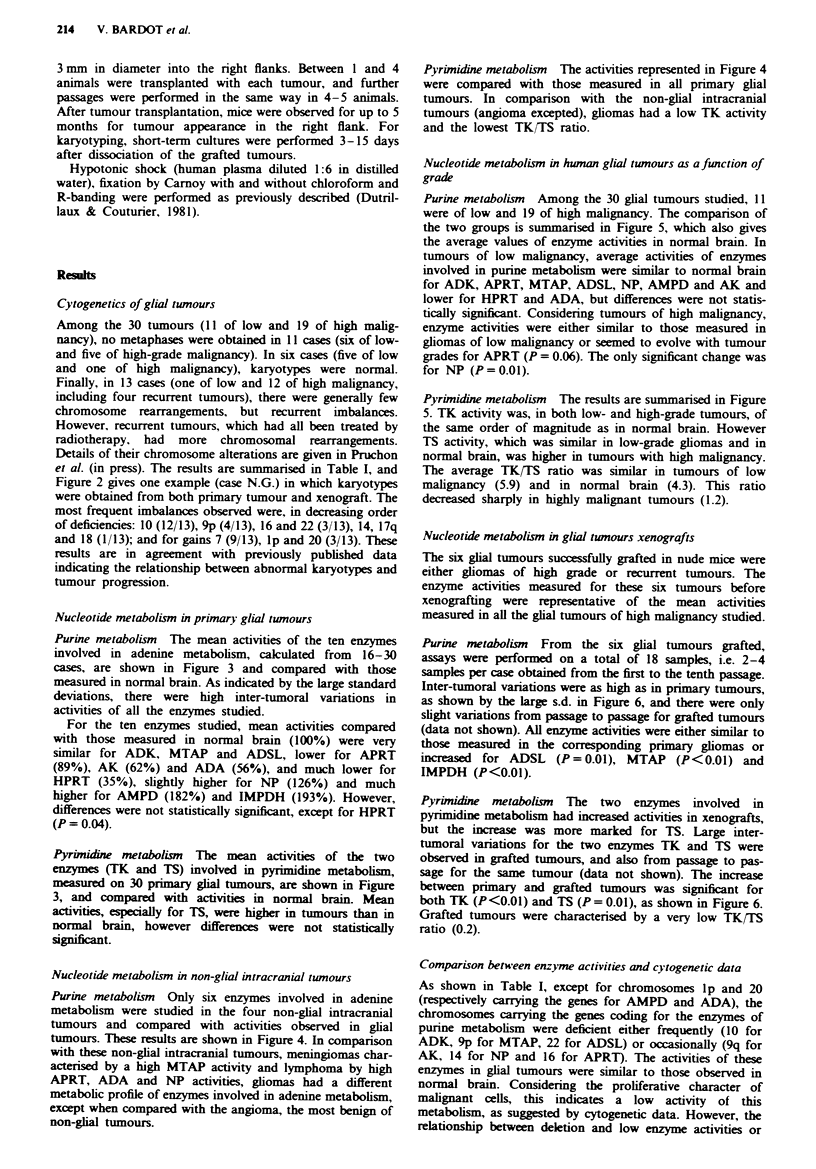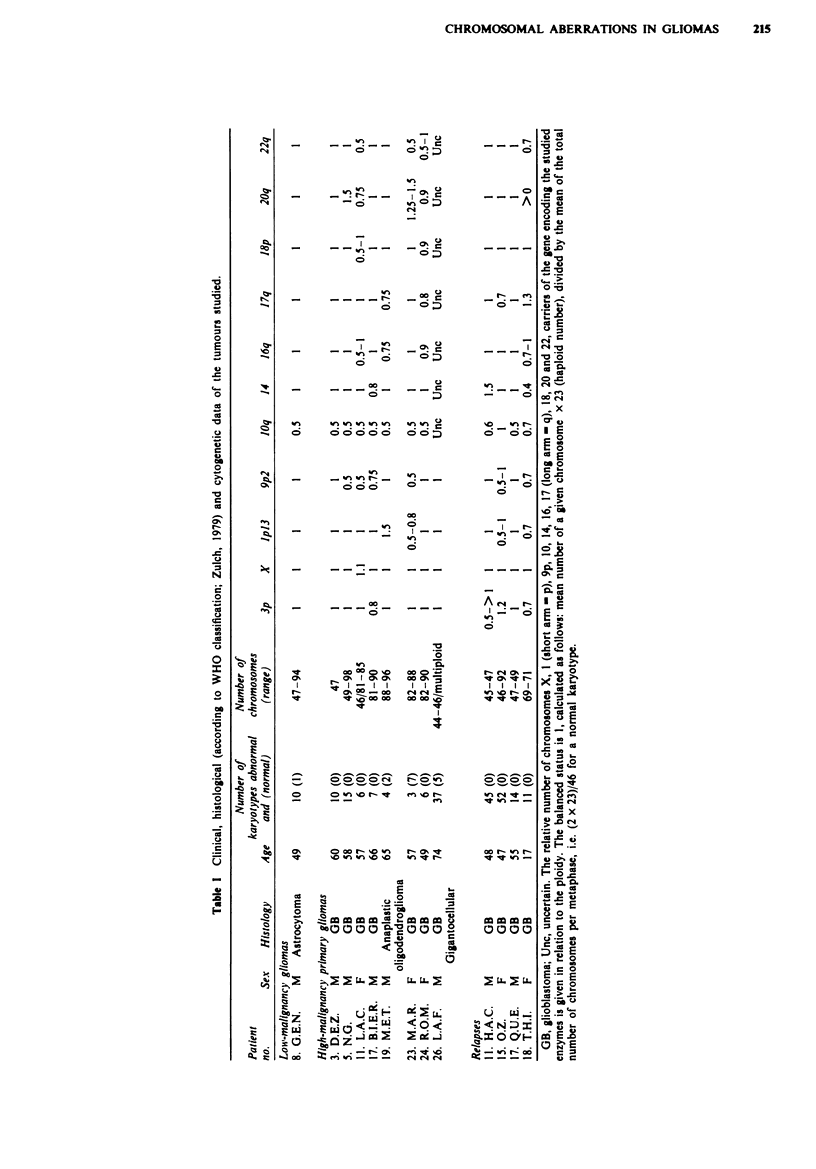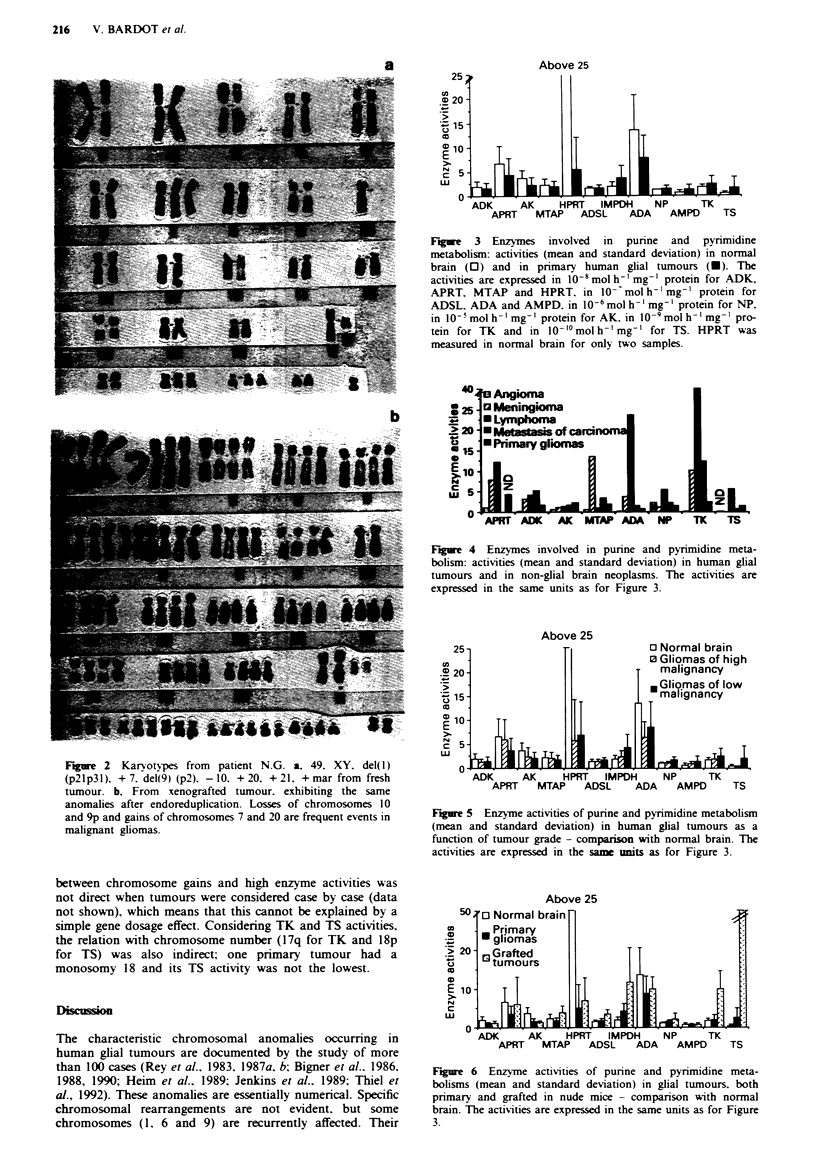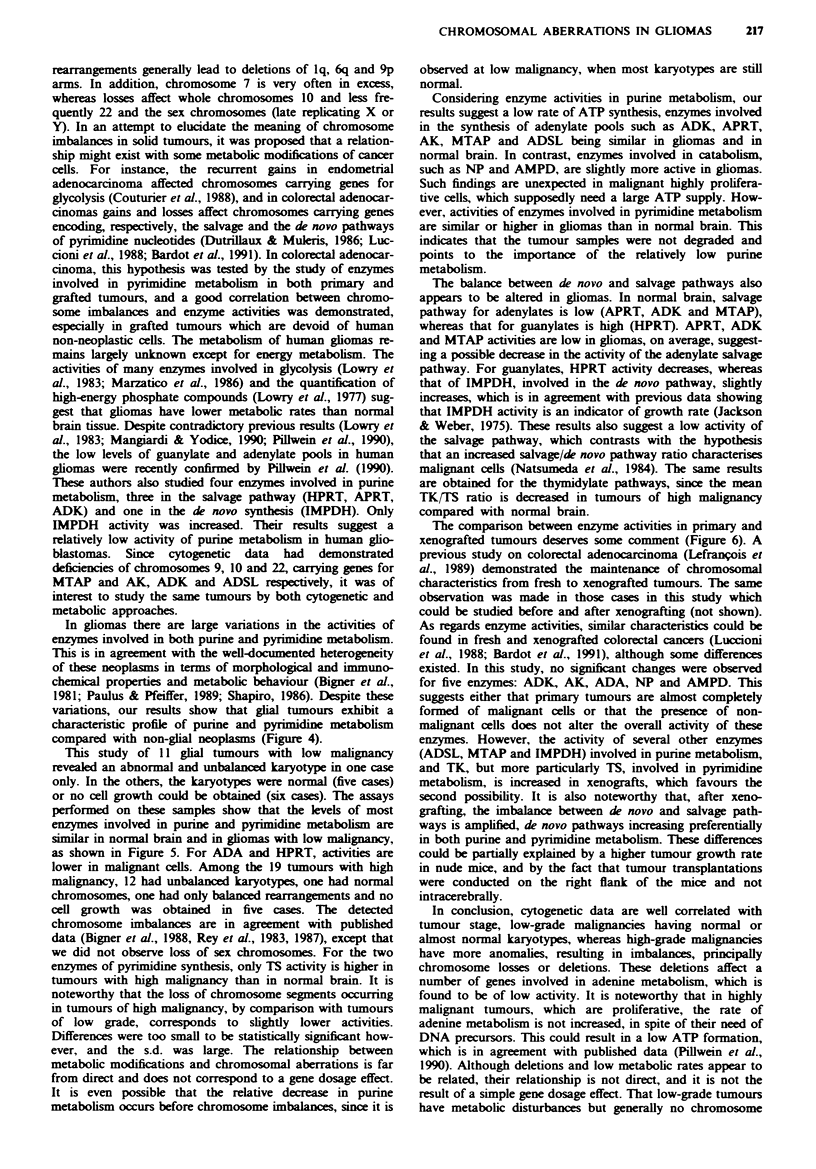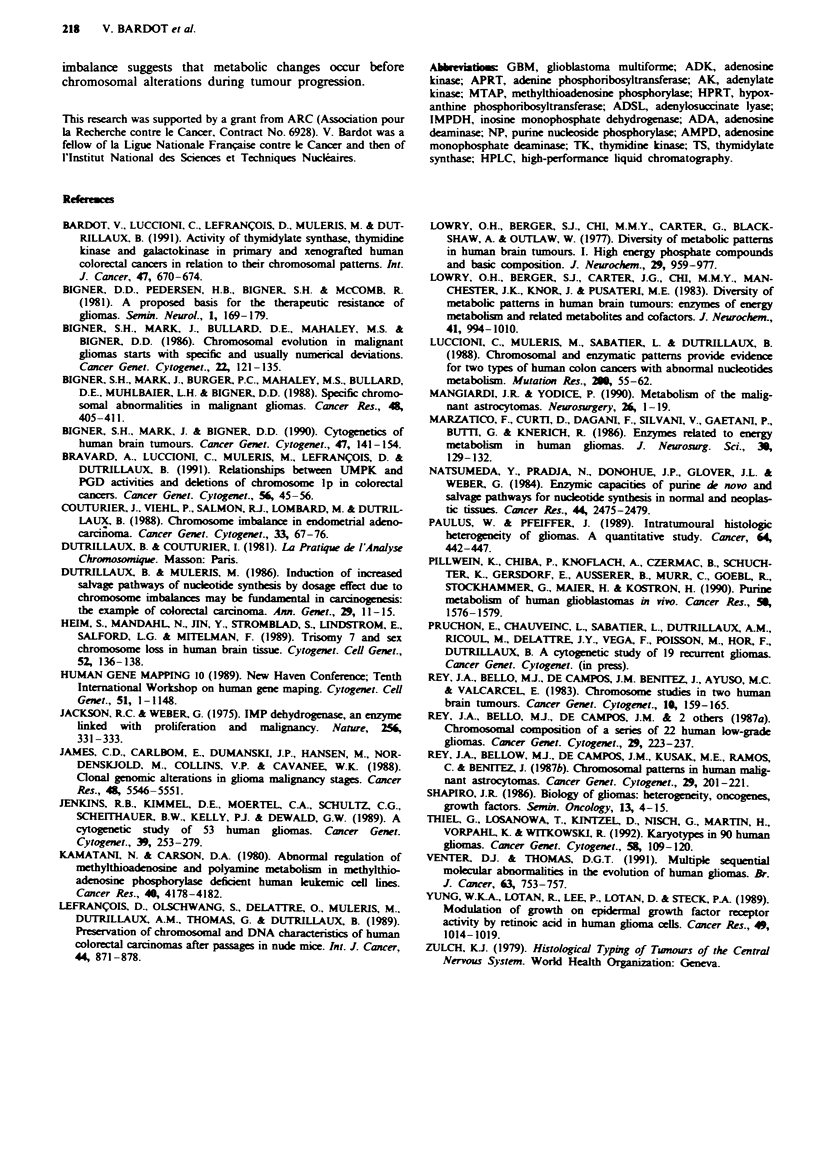# Purine and pyrimidine metabolism in human gliomas: relation to chromosomal aberrations.

**DOI:** 10.1038/bjc.1994.282

**Published:** 1994-08

**Authors:** V. Bardot, A. M. Dutrillaux, J. Y. Delattre, F. Vega, M. Poisson, B. Dutrillaux, C. Luccioni

**Affiliations:** CEA, DSV/DPTE/LCG, Fontenay-aux-Roses, France.

## Abstract

**Images:**


Br. J. Cancer (1994), 70, 212-218

© Macmillan Press Ltd., 1994

### Purine and pyrimidine metabolism in human gliomas: relation to chromosomal aberrations

V. Bardot[1], A.M. Dutrillaux[2], J.Y. Delattre[3], F. Vega[3], M. Poisson[3], B. Dutrillaux[1,2] & C. Luccioni[1]

[1]*CEA, DSV/DPTE/LCG, BP no. 6, 92 265 Fontenay-aux-Roses, cedex, France;* [2]*URA 620, CNRS Institut Curie, Section de Biologie, 26 rue d'Ulm, 75 231 Paris cedex 05, France;* [3]*Clinique Neurologique, Hôpital de la Salpétrière, 47 boulevard de l'Hôpital, 75 651 Paris cedex 13, France.*

**Summary** Chromosomal aberrations in human gliomas are principally numerical. In tumours of low malignancy, karyotypes are frequently normal, but occasionally an excess of chromosome 7 and a loss of sex chromosome are observed. In highly malignant tumours, the most frequent aberrations are gain of chromosome 7, loss of chromosome 10 and less frequently losses or deletions of chromosomes 9, 22, 6, 13 and 14 or gains of chromosomes 19 and 20. To understand the meaning of these chromosome imbalances, the relationships between chromosome abnormalities and metabolic disturbances were studied. The losses or deletions observed affected principally chromosomes carrying genes encoding enzymes involved in purine metabolism. The activities of ten enzymes were measured: adenosine kinase, adenine phosphoribosyltransferase, adenylate kinase, methylthioadenosine phosphorylase, hypoxanthine phosphoribosyltransferase, adenylosuccinate lyase, inosine monophosphate dehydrogenase, adenosine deaminase, nucleoside phosphorylase and adenosine monophosphate deaminase. In parallel, two enzymes involved in pyrimidine metabolism, thymidine kinase and thymidylate synthase (TS), were studied. The activities of all these enzymes were measured on samples from 30 human primary glial tumours with low or high malignancy, six xenografted tumours at different passages, four portions of normal brain tissue and four non-glial brain neoplasms. As suggested by cytogenetic data, the enzymatic results showed a relatively low activity of purine metabolism in glial tumours when compared with normal brain and non-glial brain neoplasms. Considering the two enzymes involved in pyrimidine metabolism, only TS had higher activity in glial tumours of high malignancy than in normal brain. In comparison with normal brain, the balance between salvage and *de novo* pathways changes in gliomas, and even more in grafted tumours, in favour of *de novo* synthesis. The relation between chromosomes and metabolic imbalances does not correspond to a simple gene dosage effect in these tumours. These data suggest that the decrease of adenosine metabolism occurs before chromosomal aberrations appear, since it is observed in tumours of low malignancy when most karyotypes are still normal, and that the *de novo* pathway increases with tumour progression.

Glial tumours are the most common primary tumours in the human central nervous system. These neoplasms form a heterogeneous group, composed of several histological subtypes, with different degrees of malignancy. Glioblastoma multiforme and high-grade astrocytomas represent more than half the cases (James *et al.*, 1988; Yung *et al.*, 1989; Venter & Thomas, 1991). Despite many attempts to characterise these tumours by biochemical, cytogenetic, immunochemical and molecular approaches, progress in therapy is limited and the prognosis for patients remains poor.

Cytogenetic analyses have been extensively performed on human gliomas (Rey *et al.*, 1983, 1987*a*, *b*; Bigner *et al.*, 1986, 1988, 1990; Heim *et al.*, 1989; Jenkins *et al.*, 1989; Thiel *et al.*, 1992). In tumours of low malignancy, the karyotype is frequently normal or may exhibit a loss of sex chromosome and a gain of chromosome 7. These two abnormalities, being observed in normal cells in culture (Rey *et al.*, 1987*a*; Heim *et al.*, 1989; Thiel *et al.*, 1992), may not necessarily characterise a malignant condition. Hence, no specific chromosomal anomaly may occur in such tumours. In highly malignant tumours, by contrast, karyotypes tend to become polyploid with an accumulation of anomalies, among which gain of chromosome 7 and loss of chromosome 10 are the most frequent. Other abnormalities, such as losses or deletions of chromosomes 9, 22, 6, 13 and 14 or gains of chromosomes 19 and 20, may be observed also, but less frequently (Rey *et al.*, 1987*b*; Bigner *et al.*, 1988, 1990; Jenkins *et al.*, 1989). In addition, a large proportion of highly malignant gliomas have double minute chromosomes (Bigner *et al.*, 1986, 1990; Thiel *et al.*, 1992).

We postulate that these recurrent chromosomal imbalances could be related to metabolic disturbances, as in human colorectal adenocarcinomas, in which activities of *de novo* and salvage pathways for pyrimidine synthesis have been related to cytogenetic anomalies (Luccioni *et al.*, 1988; Bardot *et al.*, 1991; Bravard *et al.*, 1991). In brain tumours, cytogenetic anomalies (especially losses) tend to involve chromosomes carrying genes encoding enzymes of adenine metabolism: *AK*1 and *AK*2 (adenylate kinase) and MTAP (methylthioadenosine phosphorylase) on chromosome 9, *ADK* (adenosine kinase) and *ATPase* (ATP synthetase mitochondrial) on chromosome 10, *ADSL* (adenylosuccinate lyase) on chromosome 22, *NP* (purine nucleoside phosphorylase) on chromosome 14 and *ADA* (adenosine deaminase) on chromosome 20 (HMG 10, 1989). This led us to search for a possible modification of adenine metabolism in these tumours by assaying the activities of ten enzymes involved in *de novo* salvage and catabolism pathways: ADK (EC 2.7.1.20), AK (EC 2.7.4.3), MTAP (EC 2.4.2.28), APRT (adenine phosphoribosyltransferase, EC 2.4.2.7), HPRT (hypoxanthine phosphoribosyltransferase, EC 2.4.2.8), ADSL (EC 4.3.2.2), IMPDH (inosine monophosphate dehydrogenase, EC 1.1.1.205), NP (EC 2.4.2.1), ADA (EC 3.5.4.4) and AMPD (adenosine monophosphate deaminase, EC 3.5.4.6). The relationship of these enzymes in the general pathway of purine metabolism is shown in Figure 1. In parallel, two enzymes involved in pyrimidine synthesis, TK (thymidine kinase, EC 2.7.1.21) and TS (thymidylate synthase, EC 2.1.1.45), were studied.

Since tumours grafted in nude mice are widely used and represent an interesting source of partially purified tumour tissue, we also studied six human gliomas growing as nude mouse xenografts.

### Materials and methods

### *Chemicals*

All chemicals were purchased from Sigma (St Louis, MO, USA). [8-$^{14}$C]adenosine, [8-$^{14}$C]adenine, [8-$^{14}$C]inosine mono-

Correspondence: V. Bardot.

Received 10 August 1993; and in revised form 13 November 1993.

**Figure 1** Purine metabolism. Bold characters indicate the enzymes studied.

phosphate and [5-$^{3}$H]deoxyuridine monophosphate (dUMP) were purchased from Amersham (Les Ulis, France), [$^{14}$C]-thymidine from Oris (Commissariat à l'Energie Atomique, Gif sur Yvette, France) and [8-$^{14}$C]5'deoxy-5'-methylthioadenosine from Moravek Biochemicals (Brea, USA).

*Origin and conservation of tumour samples*

Enzyme assays for purine and pyrimidine metabolism were performed on 30 glial tumours from adult patients and on four samples of normal brain, taken far from tumour tissue. In parallel, these enzymes were also studied in four diverse benign or malignant non-glial human brain tumours (one angioma, one meningioma, one lymphoma and one metastasis of carcinoma). All samples were obtained after surgical resection at the Pitie Salpetriere Hospital (Paris, France). Glial tumours were classified as of low malignancy (11 cases) in the case of histological grades I and II astrocytomas and oligodendrogliomas and as highly malignant (19 patients, including five who had relapsed) in the case of histological grades III and IV astrocytomas, oligodendrogliomas, anaplastic tumours and glioblastoma multiforme (GBM).

Tissue samples were taken in the proliferating region of the tumours: one part for cytogenetic studies, one part for transplantation into nude mice and one part frozen in liquid nitrogen for further enzymatic studies. In the case of six highly malignant glial tumours, studies were performed on tumours grafted in nude mice, from passage 1 to passage 10.

*Preparation of homogenates*

Samples were homogenised in Tris-HCl buffer 50 mM, sucrose 250 mM, Triton X-100 0.1%, pH 7.5 (1/1, w/v). Homogenates were then centrifuged at 20,000 $g$ for 1 h and supernatants were stored as aliquots at $-80$°C for all further enzymatic measurements.

*Enzyme assays*

Purine metabolism, including the ten enzymes studied, is depicted schematically in Figure 1. The activities of ADK, APRT, AK, HPRT, ADSL, ADA, NP and AMPD were determined according to methods to be described elsewhere. MTAP activity was determined by measuring the formation of adenine from [8-$^{14}$C]methylthioadenosine, according to a method described by Kamatani and Carson (1980). The final concentrations of the reaction mixture were 100 mM phosphate buffer, pH 7.4, 35 mM potassium hydrogen phosphate, 0.05 mM [8-$^{14}$C]5'-deoxy-5'-methylthioadenosine (4.6 Ci mol$^{-1}$). The reaction mixture was incubated for 45 min at 37°C and the reaction stopped by heating at 100°C for 2 min. Samples were then centrifuged at 15,000 $g$ for 20 min, before quantification of products and substrates by a high-performance liquid chromatographic (HPLC) method. The separation was effected on a reversed-phase silica column, $C_{18}$ Novapak (Waters). The mobile phase was composed of 15.5% methanol and 2% 500 mM phosphate buffer with an apparent pH of 6. The isocratic elution was run at a flow rate of 1 ml min$^{-1}$. TK activity (total activity) was determined by a radiochemical assay (Bardot *et al*., 1991). TS activity was determined by measuring the formation of tritiated water from [5-$^{3}$H]dUMP (Bardot *et al*., 1991).

For each enzyme assay, linearity with time and protein content was verified. Liver extracts from newborn rats were run in parallel as standard and all values were read against a blank without cellular extract. All assays were performed in duplicate and results were normalised to the protein content for each sample. Protein content was estimated using a Bio-Rad kit (Richmond, CA, USA) with albumin as standard. All enzymatic assays were adapted from previously published methods, to use microquantities of tumour samples.

The Mann-Whitney $U$-test was used for comparing the different groups of samples: primary tumours versus normal brain tissue, tumours of low grade versus tumours of high grade and grafted tumours versus the corresponding primary tumours.

*Cytogenetic studies*

For direct karyotyping, fresh human tumours were minced with scissors and seeded into medium F12 (Gibco) containing 20% fetal calf serum and epidermal growth factor (Sigma) (0.03 mg l$^{-1}$). Metaphases were obtained after 1-3 weeks' culture.

Athymic 5-week-old female nude mice (*nu*/*nu*, Swiss genetic background) were transplanted with pieces of approximately

3 mm in diameter into the right flanks. Between 1 and 4 animals were transplanted with each tumour, and further passages were performed in the same way in 4–5 animals. After tumour transplantation, mice were observed for up to 5 months for tumour appearance in the right flank. For karyotyping, short-term cultures were performed 3–15 days after dissociation of the grafted tumours.

Hypotonic shock (human plasma diluted 1:6 in distilled water), fixation by Carnoy with and without chloroform and R-banding were performed as previously described (Dutrillaux & Couturier, 1981).

### Results

*Cytogenetics of glial tumours*

Among the 30 tumours (11 of low and 19 of high malignancy), no metaphases were obtained in 11 cases (six of low- and five of high-grade malignancy). In six cases (five of low and one of high malignancy), karyotypes were normal. Finally, in 13 cases (one of low and 12 of high malignancy, including four recurrent tumours), there were generally few chromosome rearrangements, but recurrent imbalances. However, recurrent tumours, which had all been treated by radiotherapy, had more chromosomal rearrangements. Details of their chromosome alterations are given in Pruchon *et al.* (in press). The results are summarised in Table I, and Figure 2 gives one example (case N.G.) in which karyotypes were obtained from both primary tumour and xenograft. The most frequent imbalances observed were, in decreasing order of deficiencies: 10 (12/13), 9p (4/13), 16 and 22 (3/13), 14, 17q and 18 (1/13); and for gains 7 (9/13), 1p and 20 (3/13). These results are in agreement with previously published data indicating the relationship between abnormal karyotypes and tumour progression.

*Nucleotide metabolism in primary glial tumours*

*Purine metabolism* The mean activities of the ten enzymes involved in adenine metabolism, calculated from 16–30 cases, are shown in Figure 3 and compared with those measured in normal brain. As indicated by the large standard deviations, there were high inter-tumoral variations in activities of all the enzymes studied.

For the ten enzymes studied, mean activities compared with those measured in normal brain (100%) were very similar for ADK, MTAP and ADSL, lower for APRT (89%), AK (62%) and ADA (56%), and much lower for HPRT (35%), slightly higher for NP (126%) and much higher for AMPD (182%) and IMPDH (193%). However, differences were not statistically significant, except for HPRT ($P = 0.04$).

*Pyrimidine metabolism* The mean activities of the two enzymes (TK and TS) involved in pyrimidine metabolism, measured on 30 primary glial tumours, are shown in Figure 3, and compared with activities in normal brain. Mean activities, especially for TS, were higher in tumours than in normal brain, however differences were not statistically significant.

*Nucleotide metabolism in non-glial intracranial tumours*

*Purine metabolism* Only six enzymes involved in adenine metabolism were studied in the four non-glial intracranial tumours and compared with activities observed in glial tumours. These results are shown in Figure 4. In comparison with these non-glial intracranial tumours, meningiomas characterised by a high MTAP activity and lymphoma by high APRT, ADA and NP activities, gliomas had a different metabolic profile of enzymes involved in adenine metabolism, except when compared with the angioma, the most benign of non-glial tumours.

*Pyrimidine metabolism* The activities represented in Figure 4 were compared with those measured in all primary glial tumours. In comparison with the non-glial intracranial tumours (angioma excepted), gliomas had a low TK activity and the lowest TK/TS ratio.

*Nucleotide metabolism in human glial tumours as a function of grade*

*Purine metabolism* Among the 30 glial tumours studied, 11 were of low and 19 of high malignancy. The comparison of the two groups is summarised in Figure 5, which also gives the average values of enzyme activities in normal brain. In tumours of low malignancy, average activities of enzymes involved in purine metabolism were similar to normal brain for ADK, APRT, MTAP, ADSL, NP, AMPD and AK and lower for HPRT and ADA, but differences were not statistically significant. Considering tumours of high malignancy, enzyme activities were either similar to those measured in gliomas of low malignancy or seemed to evolve with tumour grades for APRT ($P = 0.06$). The only significant change was for NP ($P = 0.01$).

*Pyrimidine metabolism* The results are summarised in Figure 5. TK activity was, in both low- and high-grade tumours, of the same order of magnitude as in normal brain. However TS activity, which was similar in low-grade gliomas and in normal brain, was higher in tumours with high malignancy. The average TK/TS ratio was similar in tumours of low malignancy (5.9) and in normal brain (4.3). This ratio decreased sharply in highly malignant tumours (1.2).

*Nucleotide metabolism in glial tumours xenografts*

The six glial tumours successfully grafted in nude mice were either gliomas of high grade or recurrent tumours. The enzyme activities measured for these six tumours before xenografting were representative of the mean activities measured in all the glial tumours of high malignancy studied.

*Purine metabolism* From the six glial tumours grafted, assays were performed on a total of 18 samples, i.e. 2–4 samples per case obtained from the first to the tenth passage. Inter-tumoral variations were as high as in primary tumours, as shown by the large s.d. in Figure 6, and there were only slight variations from passage to passage for grafted tumours (data not shown). All enzyme activities were either similar to those measured in the corresponding primary gliomas or increased for ADSL ($P = 0.01$), MTAP ($P < 0.01$) and IMPDH ($P < 0.01$).

*Pyrimidine metabolism* The two enzymes involved in pyrimidine metabolism had increased activities in xenografts, but the increase was more marked for TS. Large inter-tumoral variations for the two enzymes TK and TS were observed in grafted tumours, and also from passage to passage for the same tumour (data not shown). The increase between primary and grafted tumours was significant for both TK ($P < 0.01$) and TS ($P = 0.01$), as shown in Figure 6. Grafted tumours were characterised by a very low TK/TS ratio (0.2).

*Comparison between enzyme activities and cytogenetic data*

As shown in Table I, except for chromosomes 1p and 20 (respectively carrying the genes for AMPD and ADA), the chromosomes carrying the genes coding for the enzymes of purine metabolism were deficient either frequently (10 for ADK, 9p for MTAP, 22 for ADSL) or occasionally (9q for AK, 14 for NP and 16 for APRT). The activities of these enzymes in glial tumours were similar to those observed in normal brain. Considering the proliferative character of malignant cells, this indicates a low activity of this metabolism, as suggested by cytogenetic data. However, the relationship between deletion and low enzyme activities or

**Table I** Clinical, histological (according to WHO classification; Zulch, 1979) and cytogenetic data of the tumours studied.

| *Patient no.* | *Sex* | *Histology* | *Age* | *Number of karyotypes abnormal and (normal)* | *Number of chromosomes (range)* | *3p* | *X* | *1p13* | *9p2* | *10q* | *14* | *16q* | *17q* | *18p* | *20q* | *22q* |
|---|---|---|---|---|---|---|---|---|---|---|---|---|---|---|---|---|
| *Low-malignancy gliomas* | | | | | | | | | | | | | | | | |
| 8. G.E.N. | M | Astrocytoma | 49 | 10 (1) | 47–94 | 1 | 1 | 1 | 1 | 0.5 | 1 | 1 | 1 | 1 | 1 | 1 |
| *High-malignancy primary gliomas* | | | | | | | | | | | | | | | | |
| 3. D.E.Z. | M | GB | 60 | 10 (0) | 47 | 1 | 1 | 1 | 1 | 0.5 | 1 | 1 | 1 | 1 | 1 | 1 |
| 5. N.G. | M | GB | 58 | 15 (0) | 49–98 | 1 | 1 | 1 | 0.5 | 0.5 | 1 | 1 | 1 | 1 | 1.5 | 1 |
| 11. L.A.C. | F | GB | 57 | 6 (0) | 46/81–85 | 1 | 1.1 | 1 | 0.5 | 0.5 | 1 | 0.5–1 | 1 | 0.5–1 | 0.75 | 0.5 |
| 17. B.I.E.R. | M | GB | 66 | 7 (0) | 81–90 | 0.8 | 1 | 1 | 0.75 | 0.5 | 0.8 | 1 | 1 | 1 | 1 | 1 |
| 19. M.E.T. | M | Anaplastic oligodendroglioma | 65 | 4 (2) | 88–96 | 1 | 1 | 1.5 | 1 | 0.5 | 1 | 0.75 | 0.75 | 1 | 1 | 1 |
| 23. M.A.R. | F | GB | 57 | 3 (7) | 82–88 | 1 | 1 | 0.5–0.8 | 0.5 | 0.5 | 1 | 1 | 1 | 1 | 1.25–1.5 | 0.5 |
| 24. R.O.M. | F | GB | 49 | 6 (0) | 82–90 | 1 | 1 | 1 | 1 | 0.5 | 1 | 0.9 | 0.8 | 0.9 | 0.9 | 0.5–1 |
| 26. L.A.F. | M | GB Gigantocellular | 74 | 37 (5) | 44–46/multiploid | 1 | 1 | 1 | 1 | Unc | Unc | Unc | Unc | Unc | Unc | Unc |
| *Relapses* | | | | | | | | | | | | | | | | |
| 11. H.A.C. | M | GB | 48 | 45 (0) | 45–47 | 0.5–>1 | 1 | 1 | 1 | 0.6 | 1.5 | 1 | 1 | 1 | 1 | 1 |
| 15. O.Z. | F | GB | 47 | 52 (0) | 46–92 | 1.2 | 1 | 0.5–1 | 0.5–1 | 1 | 1 | 1 | 0.7 | 1 | 1 | 1 |
| 17. Q.U.E. | M | GB | 55 | 14 (0) | 47–49 | 1 | 1 | 1 | 1 | 0.5 | 1 | 1 | 1 | 1 | 1 | 1 |
| 18. T.H.I. | F | GB | 17 | 11 (0) | 69–71 | 0.7 | 1 | 0.7 | 0.7 | 0.7 | 0.4 | 0.7–1 | 1.3 | 1 | >0 | 0.7 |

GB, glioblastoma; Unc, uncertain. The relative number of chromosomes X, 1 (short arm = p), 9p, 10, 14, 16, 17 (long arm = q), 18, 20 and 22, carriers of the gene encoding the studied enzymes is given in relation to the ploidy. The balanced status is 1, calculated as follows: mean number of a given chromosome × 23 (haploid number), divided by the mean of the total number of chromosomes per metaphase, i.e. (2 × 23)/46 for a normal karyotype.

**Figure 2** Karyotypes from patient N.G. **a**, 49, XY, del(1) (p21p31), +7, del(9) (p2), −10, +20, +21, +mar from fresh tumour. **b**, From xenografted tumour, exhibiting the same anomalies after endoreduplication. Losses of chromosomes 10 and 9p and gains of chromosomes 7 and 20 are frequent events in malignant gliomas.

between chromosome gains and high enzyme activities was not direct when tumours were considered case by case (data not shown), which means that this cannot be explained by a simple gene dosage effect. Considering TK and TS activities, the relation with chromosome number (17q for TK and 18p for TS) was also indirect; one primary tumour had a monosomy 18 and its TS activity was not the lowest.

### Discussion

The characteristic chromosomal anomalies occurring in human glial tumours are documented by the study of more than 100 cases (Rey *et al.*, 1983, 1987*a*, *b*; Bigner *et al.*, 1986, 1988, 1990; Heim *et al.*, 1989; Jenkins *et al.*, 1989; Thiel *et al.*, 1992). These anomalies are essentially numerical. Specific chromosomal rearrangements are not evident, but some chromosomes (1, 6 and 9) are recurrently affected. Their

**Figure 3** Enzymes involved in purine and pyrimidine metabolism: activities (mean and standard deviation) in normal brain (□) and in primary human glial tumours (■). The activities are expressed in $10^{-8}$ mol $h^{-1}$ $mg^{-1}$ protein for ADK, APRT, MTAP and HPRT, in $10^{-7}$ mol $h^{-1}$ $mg^{-1}$ protein for ADSL, ADA and AMPD, in $10^{-6}$ mol $h^{-1}$ $mg^{-1}$ protein for NP, in $10^{-5}$ mol $h^{-1}$ $mg^{-1}$ protein for AK, in $10^{-9}$ mol $h^{-1}$ $mg^{-1}$ protein for TK and in $10^{-10}$ mol $h^{-1}$ $mg^{-1}$ for TS. HPRT was measured in normal brain for only two samples.

**Figure 4** Enzymes involved in purine and pyrimidine metabolism: activities (mean and standard deviation) in human glial tumours and in non-glial brain neoplasms. The activities are expressed in the same units as for Figure 3.

**Figure 5** Enzyme activities of purine and pyrimidine metabolism (mean and standard deviation) in human glial tumours as a function of tumour grade – comparison with normal brain. The activities are expressed in the same units as for Figure 3.

**Figure 6** Enzyme activities of purine and pyrimidine metabolisms (mean and standard deviation) in glial tumours, both primary and grafted in nude mice – comparison with normal brain. The activities are expressed in the same units as for Figure 3.

rearrangements generally lead to deletions of 1q, 6q and 9p arms. In addition, chromosome 7 is very often in excess, whereas losses affect whole chromosomes 10 and less frequently 22 and the sex chromosomes (late replicating X or Y). In an attempt to elucidate the meaning of chromosome imbalances in solid tumours, it was proposed that a relationship might exist with some metabolic modifications of cancer cells. For instance, the recurrent gains in endometrial adenocarcinoma affected chromosomes carrying genes for glycolysis (Couturier *et al*., 1988), and in colorectal adenocarcinomas gains and losses affect chromosomes carrying genes encoding, respectively, the salvage and the *de novo* pathways of pyrimidine nucleotides (Dutrillaux & Muleris, 1986; Luccioni *et al*., 1988; Bardot *et al*., 1991). In colorectal adenocarcinoma, this hypothesis was tested by the study of enzymes involved in pyrimidine metabolism in both primary and grafted tumours, and a good correlation between chromosome imbalances and enzyme activities was demonstrated, especially in grafted tumours which are devoid of human non-neoplastic cells. The metabolism of human gliomas remains largely unknown except for energy metabolism. The activities of many enzymes involved in glycolysis (Lowry *et al*., 1983; Marzatico *et al*., 1986) and the quantification of high-energy phosphate compounds (Lowry *et al*., 1977) suggest that gliomas have lower metabolic rates than normal brain tissue. Despite contradictory previous results (Lowry *et al*., 1983; Mangiardi & Yodice, 1990; Pillwein *et al*., 1990), the low levels of guanylate and adenylate pools in human gliomas were recently confirmed by Pillwein *et al*. (1990). These authors also studied four enzymes involved in purine metabolism, three in the salvage pathway (HPRT, APRT, ADK) and one in the *de novo* synthesis (IMPDH). Only IMPDH activity was increased. Their results suggest a relatively low activity of purine metabolism in human glioblastomas. Since cytogenetic data had demonstrated deficiencies of chromosomes 9, 10 and 22, carrying genes for MTAP and AK, ADK and ADSL respectively, it was of interest to study the same tumours by both cytogenetic and metabolic approaches.

In gliomas there are large variations in the activities of enzymes involved in both purine and pyrimidine metabolism. This is in agreement with the well-documented heterogeneity of these neoplasms in terms of morphological and immunochemical properties and metabolic behaviour (Bigner *et al*., 1981; Paulus & Pfeiffer, 1989; Shapiro, 1986). Despite these variations, our results show that glial tumours exhibit a characteristic profile of purine and pyrimidine metabolism compared with non-glial neoplasms (Figure 4).

This study of 11 glial tumours with low malignancy revealed an abnormal and unbalanced karyotype in one case only. In the others, the karyotypes were normal (five cases) or no cell growth could be obtained (six cases). The assays performed on these samples show that the levels of most enzymes involved in purine and pyrimidine metabolism are similar in normal brain and in gliomas with low malignancy, as shown in Figure 5. For ADA and HPRT, activities are lower in malignant cells. Among the 19 tumours with high malignancy, 12 had unbalanced karyotypes, one had normal chromosomes, one had only balanced rearrangements and no cell growth was obtained in five cases. The detected chromosome imbalances are in agreement with published data (Bigner *et al*., 1988, Rey *et al*., 1983, 1987), except that we did not observe loss of sex chromosomes. For the two enzymes of pyrimidine synthesis, only TS activity is higher in tumours with high malignancy than in normal brain. It is noteworthy that the loss of chromosome segments occurring in tumours of high malignancy, by comparison with tumours of low grade, corresponds to slightly lower activities. Differences were too small to be statistically significant however, and the s.d. was large. The relationship between metabolic modifications and chromosomal aberrations is far from direct and does not correspond to a gene dosage effect. It is even possible that the relative decrease in purine metabolism occurs before chromosome imbalances, since it is observed at low malignancy, when most karyotypes are still normal.

Considering enzyme activities in purine metabolism, our results suggest a low rate of ATP synthesis, enzymes involved in the synthesis of adenylate pools such as ADK, APRT, AK, MTAP and ADSL being similar in gliomas and in normal brain. In contrast, enzymes involved in catabolism, such as NP and AMPD, are slightly more active in gliomas. Such findings are unexpected in malignant highly proliferative cells, which supposedly need a large ATP supply. However, activities of enzymes involved in pyrimidine metabolism are similar or higher in gliomas than in normal brain. This indicates that the tumour samples were not degraded and points to the importance of the relatively low purine metabolism.

The balance between *de novo* and salvage pathways also appears to be altered in gliomas. In normal brain, salvage pathway for adenylates is low (APRT, ADK and MTAP), whereas that for guanylates is high (HPRT). APRT, ADK and MTAP activities are low in gliomas, on average, suggesting a possible decrease in the activity of the adenylate salvage pathway. For guanylates, HPRT activity decreases, whereas that of IMPDH, involved in the *de novo* pathway, slightly increases, which is in agreement with previous data showing that IMPDH activity is an indicator of growth rate (Jackson & Weber, 1975). These results also suggest a low activity of the salvage pathway, which contrasts with the hypothesis that an increased salvage/*de novo* pathway ratio characterises malignant cells (Natsumeda *et al*., 1984). The same results are obtained for the thymidylate pathways, since the mean TK/TS ratio is decreased in tumours of high malignancy compared with normal brain.

The comparison between enzyme activities in primary and xenografted tumours deserves some comment (Figure 6). A previous study on colorectal adenocarcinoma (Lefrançois *et al*., 1989) demonstrated the maintenance of chromosomal characteristics from fresh to xenografted tumours. The same observation was made in those cases in this study which could be studied before and after xenografting (not shown). As regards enzyme activities, similar characteristics could be found in fresh and xenografted colorectal cancers (Luccioni *et al*., 1988; Bardot *et al*., 1991), although some differences existed. In this study, no significant changes were observed for five enzymes: ADK, AK, ADA, NP and AMPD. This suggests either that primary tumours are almost completely formed of malignant cells or that the presence of non-malignant cells does not alter the overall activity of these enzymes. However, the activity of several other enzymes (ADSL, MTAP and IMPDH) involved in purine metabolism, and TK, but more particularly TS, involved in pyrimidine metabolism, is increased in xenografts, which favours the second possibility. It is also noteworthy that, after xenografting, the imbalance between *de novo* and salvage pathways is amplified, *de novo* pathways increasing preferentially in both purine and pyrimidine metabolism. These differences could be partially explained by a higher tumour growth rate in nude mice, and by the fact that tumour transplantations were conducted on the right flank of the mice and not intracerebrally.

In conclusion, cytogenetic data are well correlated with tumour stage, low-grade malignancies having normal or almost normal karyotypes, whereas high-grade malignancies have more anomalies, resulting in imbalances, principally chromosome losses or deletions. These deletions affect a number of genes involved in adenine metabolism, which is found to be of low activity. It is noteworthy that in highly malignant tumours, which are proliferative, the rate of adenine metabolism is not increased, in spite of their need of DNA precursors. This could result in a low ATP formation, which is in agreement with published data (Pillwein *et al*., 1990). Although deletions and low metabolic rates appear to be related, their relationship is not direct, and it is not the result of a simple gene dosage effect. That low-grade tumours have metabolic disturbances but generally no chromosome imbalance suggests that metabolic changes occur before chromosomal alterations during tumour progression.

This research was supported by a grant from ARC (Association pour la Recherche contre le Cancer, Contract No. 6928). V. Bardot was a fellow of la Ligue Nationale Française contre le Cancer and then of l'Institut National des Sciences et Techniques Nucléaires.

**Abbreviations:** GBM, glioblastoma multiforme; ADK, adenosine kinase; APRT, adenine phosphoribosyltransferase; AK, adenylate kinase; MTAP, methylthioadenosine phosphorylase; HPRT, hypoxanthine phosphoribosyltransferase; ADSL, adenylosuccinate lyase; IMPDH, inosine monophosphate dehydrogenase; ADA, adenosine deaminase; NP, purine nucleoside phosphorylase; AMPD, adenosine monophosphate deaminase; TK, thymidine kinase; TS, thymidylate synthase; HPLC, high-performance liquid chromatography.

### References

BARDOT, V., LUCCIONI, C., LEFRANÇOIS, D., MULERIS, M. & DUTRILLAUX, B. (1991). Activity of thymidylate synthase, thymidine kinase and galactokinase in primary and xenografted human colorectal cancers in relation to their chromosomal patterns. *Int. J. Cancer*, **47**, 670-674.

BIGNER, D.D., PEDERSEN, H.B., BIGNER, S.H. & MCCOMB, R. (1981). A proposed basis for the therapeutic resistance of gliomas. *Semin. Neurol.*, **1**, 169-179.

BIGNER, S.H., MARK, J., BULLARD, D.E., MAHALEY, M.S. & BIGNER, D.D. (1986). Chromosomal evolution in malignant gliomas starts with specific and usually numerical deviations. *Cancer Genet. Cytogenet.*, **22**, 121-135.

BIGNER, S.H., MARK, J., BURGER, P.C., MAHALEY, M.S., BULLARD, D.E., MUHLBAIER, L.H. & BIGNER, D.D. (1988). Specific chromosomal abnormalities in malignant gliomas. *Cancer Res.*, **48**, 405-411.

BIGNER, S.H., MARK, J. & BIGNER, D.D. (1990). Cytogenetics of human brain tumours. *Cancer Genet. Cytogenet.*, **47**, 141-154.

BRAVARD, A., LUCCIONI, C., MULERIS, M., LEFRANÇOIS, D. & DUTRILLAUX, B. (1991). Relationships between UMPK and PGD activities and deletions of chromosome 1p in colorectal cancers. *Cancer Genet. Cytogenet.*, **56**, 45-56.

COUTURIER, J., VIEHL, P., SALMON, R.J., LOMBARD, M. & DUTRILLAUX, B. (1988). Chromosome imbalance in endometrial adenocarcinoma. *Cancer Genet. Cytogenet.*, **33**, 67-76.

DUTRILLAUX, B. & COUTURIER, I. (1981). *La Pratique de l'Analyse Chromosomique*. Masson: Paris.

DUTRILLAUX, B. & MULERIS, M. (1986). Induction of increased salvage pathways of nucleotide synthesis by dosage effect due to chromosome imbalances may be fundamental in carcinogenesis: the example of colorectal carcinoma. *Ann. Genet.*, **29**, 11-15.

HEIM, S., MANDAHL, N., JIN, Y., STROMBLAD, S., LINDSTROM, E., SALFORD, L.G. & MITELMAN, F. (1989). Trisomy 7 and sex chromosome loss in human brain tissue. *Cytogenet. Cell Genet.*, **52**, 136-138.

HUMAN GENE MAPPING 10 (1989). New Haven Conference; Tenth International Workshop on human gene maping. *Cytogenet. Cell Genet.*, **51**, 1-1148.

JACKSON, R.C. & WEBER, G. (1975). IMP dehydrogenase, an enzyme linked with proliferation and malignancy. *Nature*, **256**, 331-333.

JAMES, C.D., CARLBOM, E., DUMANSKI, J.P., HANSEN, M., NORDENSKJOLD, M., COLLINS, V.P. & CAVANEE, W.K. (1988). Clonal genomic alterations in glioma malignancy stages. *Cancer Res.*, **48**, 5546-5551.

JENKINS, R.B., KIMMEL, D.E., MOERTEL, C.A., SCHULTZ, C.G., SCHEITHAUER, B.W., KELLY, P.J. & DEWALD, G.W. (1989). A cytogenetic study of 53 human gliomas. *Cancer Genet. Cytogenet.*, **39**, 253-279.

KAMATANI, N. & CARSON, D.A. (1980). Abnormal regulation of methylthioadenosine and polyamine metabolism in methylthioadenosine phosphorylase deficient human leukemic cell lines. *Cancer Res.*, **40**, 4178-4182.

LEFRANÇOIS, D., OLSCHWANG, S., DELATTRE, O., MULERIS, M., DUTRILLAUX, A.M., THOMAS, G. & DUTRILLAUX, B. (1989). Preservation of chromosomal and DNA characteristics of human colorectal carcinomas after passages in nude mice. *Int. J. Cancer*, **44**, 871-878.

LOWRY, O.H., BERGER, S.J., CHI, M.M.Y., CARTER, G., BLACKSHAW, A. & OUTLAW, W. (1977). Diversity of metabolic patterns in human brain tumours. I. High energy phosphate compounds and basic composition. *J. Neurochem.*, **29**, 959-977.

LOWRY, O.H., BERGER, S.J., CARTER, J.G., CHI, M.M.Y., MANCHESTER, J.K., KNOR, J. & PUSATERI, M.E. (1983). Diversity of metabolic patterns in human brain tumours: enzymes of energy metabolism and related metabolites and cofactors. *J. Neurochem.*, **41**, 994-1010.

LUCCIONI, C., MULERIS, M., SABATIER, L. & DUTRILLAUX, B. (1988). Chromosomal and enzymatic patterns provide evidence for two types of human colon cancers with abnormal nucleotides metabolism. *Mutation Res.*, **200**, 55-62.

MANGIARDI, J.R. & YODICE, P. (1990). Metabolism of the malignant astrocytomas. *Neurosurgery*, **26**, 1-19.

MARZATICO, F., CURTI, D., DAGANI, F., SILVANI, V., GAETANI, P., BUTTI, G. & KNERICH, R. (1986). Enzymes related to energy metabolism in human gliomas. *J. Neurosurg. Sci.*, **30**, 129-132.

NATSUMEDA, Y., PRADJA, N., DONOHUE, J.P., GLOVER, J.L. & WEBER, G. (1984). Enzymic capacities of purine *de novo* and salvage pathways for nucleotide synthesis in normal and neoplastic tissues. *Cancer Res.*, **44**, 2475-2479.

PAULUS, W. & PFEIFFER, J. (1989). Intratumoural histologic heterogeneity of gliomas. A quantitative study. *Cancer*, **64**, 442-447.

PILLWEIN, K., CHIBA, P., KNOFLACH, A., CZERMAC, B., SCHUCHTER, K., GERSDORF, E., AUSSERER, B., MURR, C., GOEBL, R., STOCKHAMMER, G., MAIER, H. & KOSTRON, H. (1990). Purine metabolism of human glioblastomas *in vivo*. *Cancer Res.*, **50**, 1576-1579.

PRUCHON, E., CHAUVEINC, L., SABATIER, L., DUTRILLAUX, A.M., RICOUL, M., DELATTRE, J.Y., VEGA, F., POISSON, M., HOR, F., DUTRILLAUX, B. A cytogenetic study of 19 recurrent gliomas. *Cancer Genet. Cytogenet.* (in press).

REY, J.A., BELLO, M.J., DE CAMPOS, J.M. BENITEZ, J., AYUSO, M.C. & VALCARCEL, E. (1983). Chromosome studies in two human brain tumours. *Cancer Genet. Cytogenet.*, **10**, 159-165.

REY, J.A., BELLO, M.J., DE CAMPOS, J.M. & 2 others (1987*a*). Chromosomal composition of a series of 22 human low-grade gliomas. *Cancer Genet. Cytogenet.*, **29**, 223-237.

REY, J.A., BELLOW, M.J., DE CAMPOS, J.M., KUSAK, M.E., RAMOS, C. & BENITEZ, J. (1987*b*). Chromosomal patterns in human malignant astrocytomas. *Cancer Genet. Cytogenet.*, **29**, 201-221.

SHAPIRO, J.R. (1986). Biology of gliomas: heterogeneity, oncogenes, growth factors. *Semin. Oncology*, **13**, 4-15.

THIEL, G., LOSANOWA, T., KINTZEL, D., NISCH, G., MARTIN, H., VORPAHL, K. & WITKOWSKI, R. (1992). Karyotypes in 90 human gliomas. *Cancer Genet. Cytogenet.*, **58**, 109-120.

VENTER, D.J. & THOMAS, D.G.T. (1991). Multiple sequential molecular abnormalities in the evolution of human gliomas. *Br. J. Cancer*, **63**, 753-757.

YUNG, W.K.A., LOTAN, R., LEE, P., LOTAN, D. & STECK, P.A. (1989). Modulation of growth on epidermal growth factor receptor activity by retinoic acid in human glioma cells. *Cancer Res.*, **49**, 1014-1019.

ZULCH, K.J. (1979). *Histological Typing of Tumours of the Central Nervous System*. World Health Organization: Geneva.